# Crystal structure of (*R*)-5-[(*R*)-3-(4-chloro­phen­yl)-5-methyl-4,5-di­hydro­isoxazol-5-yl]-2-methyl­cyclo­hex-2-enone

**DOI:** 10.1107/S2056989020001991

**Published:** 2020-02-18

**Authors:** Ali Oubella, My Youssef Ait Itto, Aziz Auhmani, Abdelkhalek Riahi, Jean-Claude Daran, Abdelwahed Auhmani

**Affiliations:** a Laboratoire de Synthése Organique et de Physico-Chimie Moléculaire, Département de Chimie, Faculté des Sciences, Semlalia BP 2390, Marrakech 40001, Morocco; b Institut de Chimie Moléculaire de Reims, CNRS UMR 7312, Bat. Europol Agr, Moulin de la Housse, UFR Sciences, BP 1039, 51687 Reims Cédex 2, France; c Laboratoire de Chimie de Coordination, 205 route de Narbonne, 31077 Toulouse Cedex 04, France

**Keywords:** isoxazole derivatives, absolute configuration, natural products, pharmaceutical activities, crystal structure

## Abstract

The title compound, C_17_H_18_ClNO_2_, was prepared and isolated as a pure diastereoisomer, using column chromatography followed by a succession of fractional crystallizations. Its exact structure was fully identified *via* ^1^H NMR and confirmed by X-ray diffraction. It is built up from a central five-membered di­hydro­isoxazole ring to which a *p*-chloro­phenyl group and a cyclo­hex-2-enone ring are attached in the 3 and 5 positions. The cyclo­hex-2-one and isoxazoline rings each exhibit an envelope conformation. The crystal packing features C—H⋯O, C—H⋯N and C—H⋯π inter­actions, which generate a three-dimensional network.

## Abstract 2

The title compound is a pure diastereoisomer built up from a central five-membered di­hydro­isoxazole ring substituted in the 3 and 5 positions by a *p*-chloro­phenyl group and a cyclo­hex-2-enone ring. The cyclo­hex-2-one and the isoxazoline rings both exhibit an envelope conformation. The crystal packing features C—H⋯O, C—H⋯N and C—H⋯π inter­actions.

## Chemical context   

In recent years, isoxazole and isoxazoline derivatives have been considered to be good drug candidates because of their broad spectrum of pharmaceutical activities, such as anti­tumoral (Kamal *et al.*, 2010 ▸), anti­bacterial (Calí *et al.*, 2004 ▸), anti­viral (Deng *et al.*, 2009 ▸) and anti-inflammatory (Pedada *et al.*, 2016 ▸). Cyclo­addition and heterocyclization reactions have been widely used as synthetic methods for obtaining isoxazoles (Nieto *et al.*, 2019 ▸). In terms of selectivity, 1,3-dipolar cyclo­addition reactions of nitrilimines with dipolarophiles, such as C=C, C=S or C=N, give high stereoselectivity (Ait Itto *et al.*, 2013 ▸), while nitrile oxides, which are less sterically hindered dipoles, lead to poor stereoselectivity (Feddouli *et al.*, 2006 ▸). This was confirmed in our recent work (Oubella *et al.*, 2019 ▸) in which the 1,3-cyclo­addition reaction of di­aryl­nitrilimines with (*R*)-carvone gave the corresponding pyrazoles isolated as the unique (3a*R*,5*R*,7a*R*) diastereoisomer, while the isoxazoles prepared with nitrile oxides were isolated as (*R*,*R*)/(*R*,*S*) diastereoisomeric mixtures with a slight predominance of (*R*, *R*). In the present work, we report the separation, identification by ^1^H NMR spectroscopy, and X-ray structural analysis of the slightly major diastereoisomer of the isoxazole obtained by the 1,3-dipolar cyclo­addition of 4-chloro­benzo­nitrile oxide with (*R*)-carvone.

## Structural commentary   

**Figure 1:**
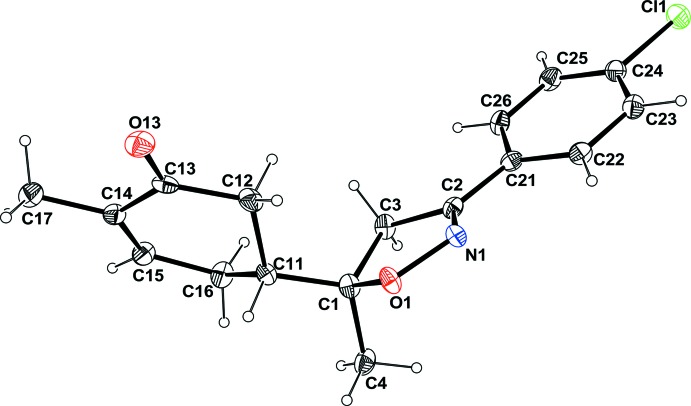
Mol­ecular structure of the title compound with the atom-numbering scheme. Displacement ellipsoids are drawn at the 50% probability level. H atoms are represented as small circles of arbitrary radii.

The title compound is built up from a central five-membered di­hydro­isoxazol ring to which a *p*-chloro­phenyl group and a cyclo­hex-2-enone ring are attached to atoms C2 and C1 at the 3 and 5 positions, respectively (Fig. 1 ▸). Atom C1 also bears a methyl group. The absolute configuration of *R*/*R* at atoms C1 and C11 were confirmed by the Flack parameter (Parsons *et al.*, 2013 ▸). This structure is closely related to the previously reported isoxazole derivative having a methyl group in place of atom Cl 3 (Oubella *et al.*, 2019 ▸). The isoxazole ring has an envelope conformation on C1 as indicated by the puckering parameters of *Q*_2_ = 0.145 (3) Å and *φ*_2_ = 138.1 (11)°. The puckering parameters for the cyclo­hexene ring, *Q* = 0.449 (3) Å, *θ* = 126.0 (4)° and *φ* = 189.2 (5)°, agree with an envelope conformation on C11. The mean plane of the isoxazole ring makes a dihedral angle of 13.4 (2)° with the C21–C26 benzene ring, whereas it makes a dihedral angle of 66.2 (1)° with the mean plane of the C11–C16 ring.
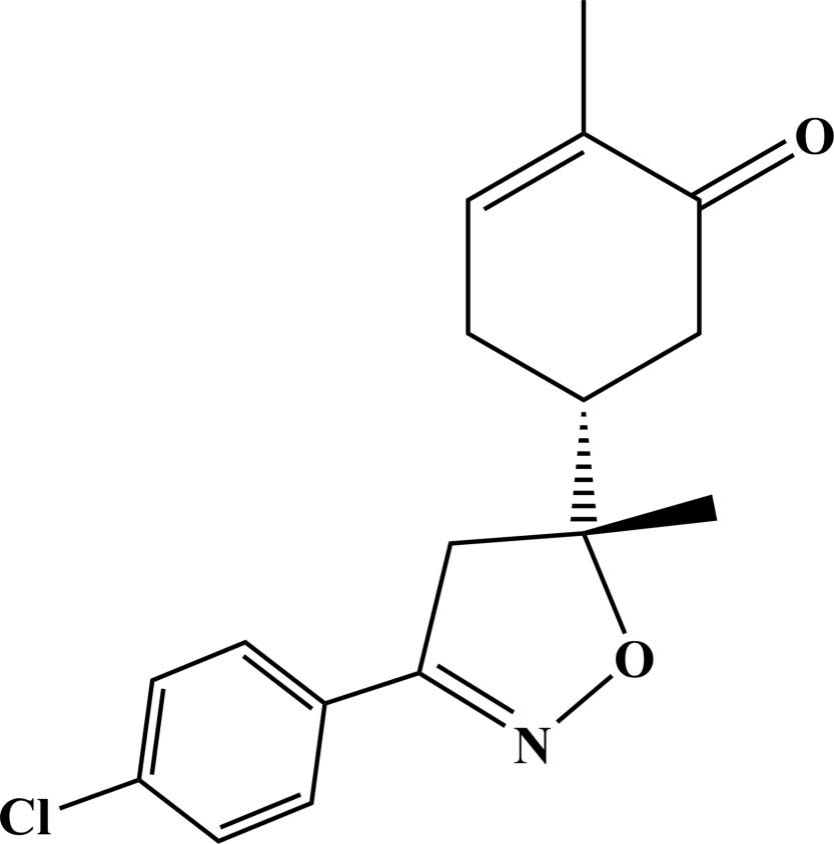


## Supra­molecular features   

**Figure 2:**
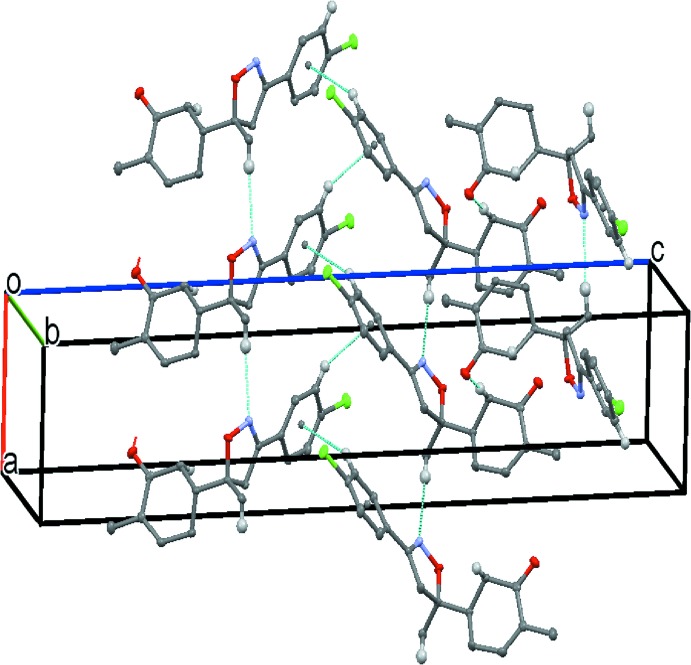
Partial packing diagram of the title compound, showing C—H⋯O, C—H⋯N and C—H⋯π inter­actions.

The packing of the structure features weak C—H⋯N and C—H⋯O inter­actions (C4—H42⋯N1^i^ and C12—H12*B*⋯O13^ii^; symmetry codes as in Table 1 ▸). The C—H⋯N inter­actions build up a linear chain along the *a-*axis direction, while the C—H⋯O inter­actions make a helical chain along the *b-*axis direction, forming a layer parallel to the *ab* plane (Fig. 2 ▸). Between the layers, a C—H⋯π inter­action is observed (C23—H23⋯*Cg*1^iii^; Table 1 ▸), where *Cg*1 is the centroid of the C21–C26 benzene ring.

## Database survey   

A search in the Cambridge Structural Database (CSD, version 5.40, update August 2019; Groom *et al.*, 2016 ▸) for organic compounds with structures containing a 2-isoxazoline ring revealed 284 hits. Introducing a methyl group on position 5 reduced this number to 15 and searching for structures containing a phenyl ring attached to position 3 gave only seven hits. A comparison of related distances and angles within the 2-isoxazoline ring shows a good agreement between all these structures, with a systematically short C2—N1 bond with lengths ranging from 1.274 to 1.285 Å, corresponding to a C=N double bond. A larger discrepancy is observed for the dihedral angle between the isoxazol mean plane and the benzene ring in the (*S*)-3-(2,6-di­chloro­phen­yl)-5-[(2,5-di­phenyl­pyrrolidin-1-yl)carbon­yl]-5-methyl-4,5-di­hydro­isoxa­zole compound (Houk *et al.*, 1984 ▸); at 66.8°, this is much larger than the value of 13.4 (2)° observed for the title compound. This larger dihedral angle is related to the occurrence of two Cl atoms in the 2 and 5 positions on the phenyl ring.

## Synthesis and crystallization   

**Figure 3:**
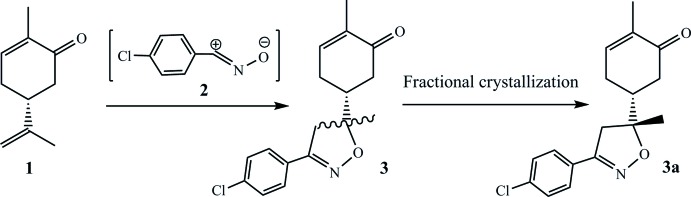
Synthesis pathway leading to the formation of the title compound, **3a**.

**Figure 4:**
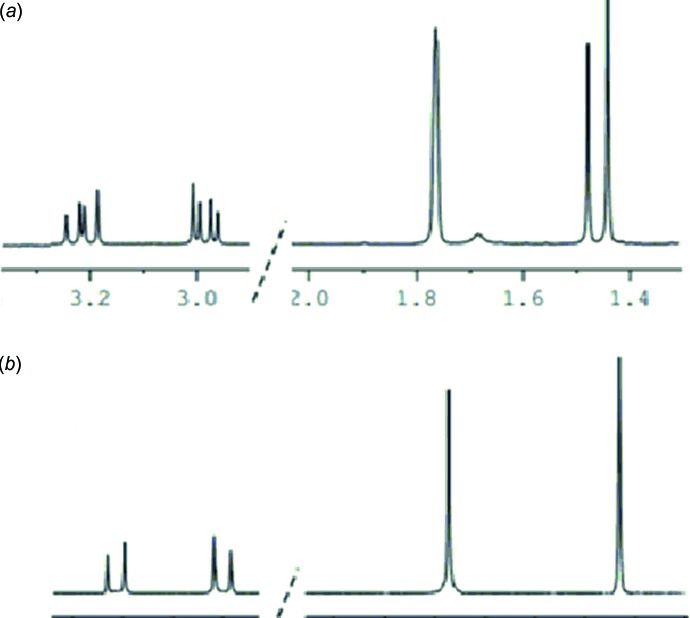
^1^H NMR spectra of (*a*) the diastereoisomeric mixture **3** and (*b*) the pure separated (5*R*,5′*R*) diastereoisomer **3a**.

As shown in Fig. 3 ▸, (*R*)-carvone, **1**, was reacted with nitrile oxide, **2**, generated *in situ* from the corresponding oxime according to our recently described methodology (Oubella *et al.*, 2019 ▸). The corresponding isoxazole, **3**, was obtained in 80% yield, as an (*R*,*R*)/(*R*,*S*) diastereoisomeric mixture. The ^1^H NMR spectrum of **3** clearly shows a splitting of both the methyl and methyl­ene groups in the α position of the newly formed stereogenic center of the isoxazole nucleus (Fig. 4 ▸ *a*). The former gave rise to two singlets at 1.44 ppm and 1.48 ppm, respectively, while the latter is seen as two pairs of doublets, one at 2.90 and 3.20 ppm (*J* = 16.9 Hz) and the other at 2.75 and 3.30 ppm (*J* = 16.7 Hz). Integrating the corresponding ^1^H NMR signals allowed us to qu­antify the ratio of the diastereoisomereric mixture as 58:42. After several attempts at separation, either by column chromatography or a series of fractional crystallizations by slow evaporation from a chloro­form solution of **3**, we managed to separate the diastereo­isomer **3a**, the title compound, as pure single crystals suitable for crystallographic analysis. Its ^1^H NMR spectrum (Fig. 4 ▸ *b*) is mainly characterized by the isoxazolic methyl group resonating as a singlet at 1.44 ppm, and the methyl­ene group appeared as two doublets at 2.90 ppm (*J* = 16.9 Hz) and 3.20 ppm (*J* = 16.9 Hz).

## Refinement   

Crystal data, data collection and structure refinement details are summarized in Table 2 ▸. All H atoms attached to C atoms were fixed geometrically and treated as riding with C—H = 0.99 Å (methyl­ene), 0.98 Å (meth­yl) or 0.95 Å (methine), and with *U*_iso_(H) = 1.2*U*_eq_(C) for methyl­ene and methine or *U*_iso_(H) = 1.5*U*_eq_(C) for methyl H atoms.

## Supplementary Material

Crystal structure: contains datablock(s) I, global. DOI: 10.1107/S2056989020001991/is5530sup1.cif

Structure factors: contains datablock(s) I. DOI: 10.1107/S2056989020001991/is5530Isup2.hkl

Supporting information file. DOI: 10.1107/S2056989020001991/is5530Isup3.cml

CCDC reference: 1983547

Additional supporting information:  crystallographic information; 3D view; checkCIF report

## Figures and Tables

**Table 1:** Hydrogen-bond geometry (Å, °) *Cg*1 is the centroid of the C21–C26 ring.

*D*—H⋯*A*	*D*—H	H⋯*A*	*D*⋯*A*	*D*—H⋯*A*
C4—H42⋯N1^i^	0.96	2.62	3.572 (4)	173
C12—H12*B*⋯O13^ii^	0.97	2.54	3.488 (4)	165
C23—H23⋯*Cg*1^iii^	0.93	2.71	3.554 (3)	151

Symmetry codes: (i) 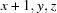; (ii) 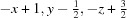; (iii) 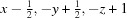.

**Table 2:** Experimental details

Crystal data	
Chemical formula	C_17_H_18_ClNO_2_
*M*_r_	303.77
Crystal system, space group	Orthorhombic, *P*2_1_2_1_2_1_
Temperature (K)	105
*a*, *b*, *c* (Å)	6.4590 (2), 7.3545 (3), 31.3436 (12)
*V* (Å^3^)	1488.91 (10)
*Z*	4
Radiation type	Mo *K*α
μ (mm^−1^)	0.26
Crystal size (mm)	0.31 × 0.26 × 0.18

Data collection	
Diffractometer	Bruker APEXII CCD
Absorption correction	Multi-scan (*SADABS*; Bruker, 2015 ▸)
*T*_min_, *T*_max_	0.694, 0.746
No. of measured, independent and observed [*I* > 2σ(*I*)] reflections	16529, 3019, 2827
*R*_int_	0.057
(sin θ/λ)_max_ (Å^−1^)	0.625

Refinement	
*R*[*F* ^2^ > 2σ(*F* ^2^)], *wR*(*F* ^2^), *S*	0.035, 0.105, 1.19
No. of reflections	3019
No. of parameters	193
H-atom treatment	H-atom parameters constrained
Δρ_max_, Δρ_min_ (e Å^−3^)	0.40, −0.23
Absolute structure	Flack *x* determined using 1100 quotients [(*I* ^+^)−(*I* ^−^)]/[(*I* ^+^)+(*I* ^−^)] (Parsons *et al.*, 2013 ▸)
Absolute structure parameter	−0.09 (4)

Computer programs: *APEX2* and *SAINT* (Bruker, 2015 ▸), *SHELXT* (Sheldrick, 2015*a* ▸), *SHELXL2016* (Sheldrick, 2015*b* ▸) and *ORTEP-3 for Windows* (Farrugia, 2012 ▸).

## supplementary crystallographic information

### Crystal data

**Table:**

C_17_H_18_ClNO_2_	*D*_x_ = 1.355 Mg m^−^^3^
*M**_r_* = 303.77	Mo *K*α radiation, λ = 0.71073 Å
Orthorhombic, *P*2_1_2_1_2_1_	Cell parameters from 5113 reflections
*a* = 6.4590 (2) Å	θ = 2.8–27.9°
*b* = 7.3545 (3) Å	µ = 0.26 mm^−^^1^
*c* = 31.3436 (12) Å	*T* = 105 K
*V* = 1488.91 (10) Å^3^	Box, colourless
*Z* = 4	0.31 × 0.26 × 0.18 mm
*F*(000) = 640	

### Data collection

**Table:**

Bruker APEXII CCD diffractometer	3019 independent reflections
Radiation source: micro-focus sealed tube	2827 reflections with *I* > 2σ(*I*)
Graphite monochromator	*R*_int_ = 0.057
φ and ω scans	θ_max_ = 26.4°, θ_min_ = 1.3°
Absorption correction: multi-scan (SADABS; Bruker, 2015)	*h* = −8→8
*T*_min_ = 0.694, *T*_max_ = 0.746	*k* = −9→9
16529 measured reflections	*l* = −38→39

### Refinement

**Table:**

Refinement on *F*^2^	Hydrogen site location: inferred from neighbouring sites
Least-squares matrix: full	H-atom parameters constrained
*R*[*F*^2^ > 2σ(*F*^2^)] = 0.035	*w* = 1/[σ^2^(*F*_o_^2^) + (0.060*P*)^2^ + 0.137*P*] where *P* = (*F*_o_^2^ + 2*F*_c_^2^)/3
*wR*(*F*^2^) = 0.105	(Δ/σ)_max_ = 0.001
*S* = 1.19	Δρ_max_ = 0.40 e Å^−^^3^
3019 reflections	Δρ_min_ = −0.23 e Å^−^^3^
193 parameters	Extinction correction: SHELXL2016 (Sheldrick, 2015*b*), Fc^*^=kFc[1+0.001xFc^2^λ^3^/sin(2θ)]^-1/4^
0 restraints	Extinction coefficient: 0.014 (4)
Primary atom site location: dual	Absolute structure: Flack *x* determined using 1100 quotients [(*I*^+^)-(*I*^-^)]/[(*I*^+^)+(*I*^-^)] (Parsons *et al.*, 2013)
Secondary atom site location: difference Fourier map	Absolute structure parameter: −0.09 (4)

### Special details

**Table:**

Geometry. All esds (except the esd in the dihedral angle between two l.s. planes) are estimated using the full covariance matrix. The cell esds are taken into account individually in the estimation of esds in distances, angles and torsion angles; correlations between esds in cell parameters are only used when they are defined by crystal symmetry. An approximate (isotropic) treatment of cell esds is used for estimating esds involving l.s. planes.

### Fractional atomic coordinates and isotropic or equivalent isotropic displacement parameters (Å^2^)

**Table:**

	*x*	*y*	*z*	*U*_iso_*/*U*_eq_	
C1	0.6484 (4)	0.6566 (4)	0.64093 (9)	0.0150 (6)	
C2	0.4125 (4)	0.4711 (4)	0.60439 (8)	0.0140 (6)	
C3	0.6216 (4)	0.4598 (4)	0.62542 (9)	0.0158 (6)	
H31	0.728644	0.425833	0.605242	0.019*	
H32	0.621273	0.374475	0.649026	0.019*	
C4	0.7886 (4)	0.7654 (4)	0.61166 (9)	0.0197 (6)	
H41	0.739550	0.755866	0.582840	0.030*	
H42	0.927089	0.718468	0.613353	0.030*	
H43	0.787990	0.890632	0.620283	0.030*	
C11	0.7057 (4)	0.6782 (4)	0.68816 (9)	0.0134 (6)	
H11	0.713927	0.808792	0.694138	0.016*	
C12	0.5419 (4)	0.5983 (4)	0.71777 (9)	0.0181 (6)	
H12A	0.411443	0.660009	0.712739	0.022*	
H12B	0.522661	0.470911	0.710757	0.022*	
C13	0.5961 (4)	0.6140 (4)	0.76455 (9)	0.0155 (6)	
C14	0.8176 (4)	0.6073 (4)	0.77632 (9)	0.0163 (6)	
C15	0.9600 (4)	0.5958 (4)	0.74575 (9)	0.0175 (6)	
H15	1.097590	0.587139	0.754279	0.021*	
C16	0.9173 (4)	0.5956 (4)	0.69867 (9)	0.0182 (6)	
H16A	0.922229	0.471584	0.688155	0.022*	
H16B	1.024450	0.664296	0.684169	0.022*	
C17	0.8694 (5)	0.6108 (4)	0.82302 (9)	0.0222 (6)	
H17A	1.016154	0.596660	0.826631	0.033*	
H17B	0.798826	0.513186	0.837226	0.033*	
H17C	0.826500	0.724750	0.835047	0.033*	
C21	0.3164 (4)	0.3268 (4)	0.57902 (8)	0.0146 (6)	
C22	0.1345 (4)	0.3588 (4)	0.55578 (9)	0.0164 (6)	
H22	0.077055	0.474733	0.555387	0.020*	
C23	0.0396 (4)	0.2195 (4)	0.53343 (9)	0.0180 (6)	
H23	−0.081403	0.240979	0.518175	0.022*	
C24	0.1273 (5)	0.0476 (4)	0.53409 (9)	0.0176 (6)	
C25	0.3067 (5)	0.0123 (4)	0.55656 (9)	0.0194 (6)	
H25	0.364066	−0.103682	0.556670	0.023*	
C26	0.3997 (4)	0.1524 (4)	0.57889 (9)	0.0181 (6)	
H26	0.520399	0.129473	0.594145	0.022*	
N1	0.3162 (3)	0.6195 (3)	0.61149 (7)	0.0152 (5)	
O1	0.4384 (3)	0.7345 (3)	0.63657 (6)	0.0161 (4)	
O13	0.4603 (3)	0.6257 (3)	0.79158 (6)	0.0220 (5)	
Cl1	0.00864 (11)	−0.12745 (10)	0.50590 (2)	0.0242 (2)	

### Atomic displacement parameters (Å^2^)

**Table:**

	*U*^11^	*U*^22^	*U*^33^	*U*^12^	*U*^13^	*U*^23^
C1	0.0094 (12)	0.0177 (14)	0.0179 (14)	0.0039 (11)	−0.0007 (11)	−0.0012 (11)
C2	0.0129 (13)	0.0170 (14)	0.0121 (13)	0.0026 (11)	0.0016 (10)	0.0016 (11)
C3	0.0132 (13)	0.0177 (14)	0.0166 (14)	0.0055 (11)	−0.0009 (11)	−0.0029 (11)
C4	0.0159 (13)	0.0272 (16)	0.0161 (14)	0.0024 (13)	−0.0020 (12)	0.0036 (12)
C11	0.0098 (12)	0.0144 (13)	0.0162 (13)	0.0015 (10)	−0.0007 (11)	−0.0021 (10)
C12	0.0111 (13)	0.0221 (15)	0.0210 (14)	−0.0027 (11)	0.0007 (11)	−0.0013 (11)
C13	0.0154 (13)	0.0098 (12)	0.0211 (14)	−0.0021 (11)	0.0037 (11)	−0.0003 (11)
C14	0.0185 (13)	0.0139 (13)	0.0164 (14)	−0.0008 (12)	0.0000 (11)	0.0009 (11)
C15	0.0119 (14)	0.0218 (14)	0.0188 (14)	0.0015 (12)	−0.0007 (11)	0.0027 (11)
C16	0.0113 (13)	0.0260 (16)	0.0172 (15)	0.0045 (12)	0.0018 (11)	−0.0002 (12)
C17	0.0230 (14)	0.0263 (16)	0.0171 (14)	−0.0038 (14)	0.0013 (12)	0.0018 (13)
C21	0.0156 (13)	0.0159 (14)	0.0123 (13)	0.0008 (11)	0.0037 (11)	0.0008 (10)
C22	0.0166 (12)	0.0152 (13)	0.0173 (13)	0.0021 (12)	0.0006 (11)	0.0004 (11)
C23	0.0167 (13)	0.0214 (14)	0.0161 (13)	0.0013 (12)	−0.0007 (11)	0.0004 (11)
C24	0.0215 (15)	0.0181 (14)	0.0131 (13)	−0.0032 (12)	0.0004 (12)	−0.0017 (11)
C25	0.0260 (15)	0.0151 (13)	0.0172 (14)	0.0031 (12)	−0.0007 (13)	0.0001 (11)
C26	0.0197 (14)	0.0191 (14)	0.0155 (14)	0.0038 (12)	−0.0032 (12)	0.0004 (11)
N1	0.0134 (10)	0.0162 (11)	0.0160 (11)	−0.0017 (11)	−0.0019 (9)	−0.0017 (10)
O1	0.0106 (9)	0.0156 (9)	0.0222 (10)	0.0035 (7)	−0.0048 (8)	−0.0048 (8)
O13	0.0191 (10)	0.0249 (10)	0.0220 (10)	−0.0008 (10)	0.0067 (8)	−0.0005 (9)
Cl1	0.0307 (4)	0.0197 (4)	0.0223 (4)	−0.0051 (3)	−0.0041 (3)	−0.0033 (3)

### Geometric parameters (Å, º)

**Table:**

C1—O1	1.478 (3)	C14—C17	1.502 (4)
C1—C4	1.517 (4)	C15—C16	1.501 (4)
C1—C11	1.534 (4)	C15—H15	0.9300
C1—C3	1.537 (4)	C16—H16A	0.9700
C2—N1	1.276 (4)	C16—H16B	0.9700
C2—C21	1.464 (4)	C17—H17A	0.9600
C2—C3	1.505 (4)	C17—H17B	0.9600
C3—H31	0.9700	C17—H17C	0.9600
C3—H32	0.9700	C21—C26	1.391 (4)
C4—H41	0.9600	C21—C22	1.402 (4)
C4—H42	0.9600	C22—C23	1.384 (4)
C4—H43	0.9600	C22—H22	0.9300
C11—C12	1.525 (4)	C23—C24	1.386 (4)
C11—C16	1.532 (3)	C23—H23	0.9300
C11—H11	0.9800	C24—C25	1.380 (4)
C12—C13	1.512 (4)	C24—Cl1	1.740 (3)
C12—H12A	0.9700	C25—C26	1.383 (4)
C12—H12B	0.9700	C25—H25	0.9300
C13—O13	1.223 (3)	C26—H26	0.9300
C13—C14	1.478 (4)	N1—O1	1.398 (3)
C14—C15	1.331 (4)		
			
O1—C1—C4	106.7 (2)	C15—C14—C17	123.3 (3)
O1—C1—C11	105.7 (2)	C13—C14—C17	117.3 (2)
C4—C1—C11	112.6 (2)	C14—C15—C16	125.5 (3)
O1—C1—C3	103.4 (2)	C14—C15—H15	117.3
C4—C1—C3	111.9 (2)	C16—C15—H15	117.2
C11—C1—C3	115.5 (2)	C15—C16—C11	112.0 (2)
N1—C2—C21	120.6 (2)	C15—C16—H16A	109.2
N1—C2—C3	114.1 (2)	C11—C16—H16A	109.2
C21—C2—C3	125.3 (2)	C15—C16—H16B	109.2
C2—C3—C1	100.8 (2)	C11—C16—H16B	109.2
C2—C3—H31	111.6	H16A—C16—H16B	107.9
C1—C3—H31	111.6	C14—C17—H17A	109.5
C2—C3—H32	111.6	C14—C17—H17B	109.5
C1—C3—H32	111.6	H17A—C17—H17B	109.5
H31—C3—H32	109.4	C14—C17—H17C	109.5
C1—C4—H41	109.5	H17A—C17—H17C	109.5
C1—C4—H42	109.5	H17B—C17—H17C	109.5
H41—C4—H42	109.5	C26—C21—C22	118.5 (3)
C1—C4—H43	109.5	C26—C21—C2	120.4 (2)
H41—C4—H43	109.5	C22—C21—C2	121.0 (2)
H42—C4—H43	109.5	C23—C22—C21	120.6 (3)
C12—C11—C16	109.6 (2)	C23—C22—H22	119.7
C12—C11—C1	112.3 (2)	C21—C22—H22	119.7
C16—C11—C1	112.4 (2)	C22—C23—C24	119.1 (3)
C12—C11—H11	107.4	C22—C23—H23	120.4
C16—C11—H11	107.4	C24—C23—H23	120.4
C1—C11—H11	107.4	C25—C24—C23	121.5 (3)
C13—C12—C11	113.6 (2)	C25—C24—Cl1	119.3 (2)
C13—C12—H12A	108.9	C23—C24—Cl1	119.2 (2)
C11—C12—H12A	108.9	C24—C25—C26	118.9 (3)
C13—C12—H12B	108.9	C24—C25—H25	120.6
C11—C12—H12B	108.9	C26—C25—H25	120.6
H12A—C12—H12B	107.7	C25—C26—C21	121.4 (3)
O13—C13—C14	121.6 (3)	C25—C26—H26	119.3
O13—C13—C12	120.7 (2)	C21—C26—H26	119.3
C14—C13—C12	117.6 (2)	C2—N1—O1	109.9 (2)
C15—C14—C13	119.4 (2)	N1—O1—C1	109.61 (19)

### Hydrogen-bond geometry (Å, º)

*Cg*1 is the centroid of the C21–C26 ring.

**Table:**

*D*—H···*A*	*D*—H	H···*A*	*D*···*A*	*D*—H···*A*
C4—H42···N1^i^	0.96	2.62	3.572 (4)	173
C12—H12*B*···O13^ii^	0.97	2.54	3.488 (4)	165
C23—H23···*Cg*1^iii^	0.93	2.71	3.554 (3)	151

Symmetry codes: (i) *x*+1, *y*, *z*; (ii) −*x*+1, *y*−1/2, −*z*+3/2; (iii) *x*−1/2, −*y*+1/2, −*z*+1.
